# Effects of single bouts of different endurance exercises with different intensities on microRNA biomarkers with and without blood flow restriction: a three-arm, randomized crossover trial

**DOI:** 10.1007/s00421-021-04786-2

**Published:** 2021-08-25

**Authors:** Johanna Sieland, Daniel Niederer, Tobias Engeroff, Lutz Vogt, Christian Troidl, Thomas Schmitz-Rixen, Winfried Banzer, Kerstin Troidl

**Affiliations:** 1 Department of Sports Medicine, Institute of Sport Sciences, Goethe University, Ginnheimer Landstraße 39, 60487 Frankfurt, Germany; 2 Department of Experimental Cardiology, Medical Faculty, Justus-Liebig-University, 35392 Giessen, Germany; 3 Department of Cardiology, Kerckhoff Heart and Thorax Center, 61231 Bad Nauheim, Germany; 4 German Center for Cardiovascular Research (DZHK), Partner Site RhineMain, Frankfurt, Germany; 5 Department of Vascular and Endovascular Surgery, University Hospital Frankfurt, Theodor-Stern-Kai 7, 60590 Frankfurt, Germany; 6 Institute for Occupational Medicine, Social Medicine and Environmental Medicine, University Hospital Frankfurt, Theodor-Stern-Kai 7, 60590 Frankfurt, Germany; 7 Department of Pharmacology, Max-Planck-Institute for Heart and Lung Research, Ludwigstrasse 43, 61231 Bad Nauheim, Germany

**Keywords:** Circulating miRNA, miR-197-3p, miR-142-5p, miR-342-3p, miR-424-5p, Blood flow restriction, Endurance training

## Abstract

**Purpose:**

Physical activity is associated with altered levels of circulating microRNAs (ci-miRNAs). Changes in miRNA expression have great potential to modulate biological pathways of skeletal muscle hypertrophy and metabolism. This study was designed to determine whether the profile of ci-miRNAs is altered after different approaches of endurance exercise.

**Methods:**

Eighteen healthy volunteers (aged 24 ± 3 years) participated this three-arm, randomized-balanced crossover study. Each arm was a single bout of treadmill-based acute endurance exercise at (1) 100% of the individual anaerobic threshold (IANS), (2) at 80% of the IANS and (3) at 80% of the IANS with blood flow restriction (BFR). Load-associated outcomes (fatigue, feeling, heart rate, and exhaustion) as well as acute effects (circulating miRNA patterns and lactate) were determined.

**Results:**

All training interventions increased the lactate concentration (LC) and heart rate (HR) (*p* < 0.001). The high-intensity intervention (HI) resulted in a higher LC than both lower intensity protocols (*p* < 0.001). The low-intensity blood flow restriction (LI-BFR) protocol led to a higher HR and higher LC than the low-intensity (LI) protocol without BFR (*p* = 0.037 and *p* = 0.003). The level of miR-142-5p and miR-197-3p were up-regulated in both interventions without BFR (*p* < 0.05). After LI exercise, the expression of miR-342-3p was up-regulated (*p* = 0.038). In LI-BFR, the level of miR-342-3p and miR-424-5p was confirmed to be up-regulated (*p* < 0.05). Three miRNAs and LC show a significant negative correlation (miR-99a-5p, *p* = 0.011, *r = −* 0.343/miR-199a-3p, *p* = 0.045, *r = − *0.274/miR-125b-5p, *p* = 0.026, *r = − *0.302). Two partial correlations (intervention partialized) showed a systematic impact of the type of exercise (LI-BFR vs. HI) (miR-99a-59: *r = − *0.280/miR-199a-3p: *r = − *0.293).

**Conclusion:**

MiRNA expression patterns differ according to type of activity. We concluded that not only the intensity of the exercise (LC) is decisive for the release of circulating miRNAs—as essential is the type of training and the oxygen supply.

## Introduction

MicroRNAs (miRNAs) are small, non-coding, single-stranded, endogenously encoded RNAs consisting of approximately 20–24 nucleotides. These polymeric molecules regulate protein abundance primarily by binding to the three prime untranslated region (3’-UTR) of coding mRNAs (Grimson et al. 2007). One miRNA can regulate the expression of hundreds of mRNAs and proteins (Lim et al. 2005; Baek et al. 2008). Therefore, miRNAs play a significant role in regulating many biological processes and affect different molecular pathways (Chen et al. 2013; Weiss and Ito 2017). For example, 142-5p is reported to be an important regulator of cellular survival (Wang et al. 2017) and miRNA-125b-5p as potential biomarker for acute ischemic stroke (Tiedt et al. 2017). In addition, several miRNAs are modulators of biological pathways in skeletal muscle hypertrophy and metabolism (McCarthy and Esser 2007; Davidsen et al. 2011). Others influence alterations of the cardiovascular system (Dong and Yang 2011; Wojciechowska et al. 2017), such as miRNA-197-3p and miRNA-424 regulate endothelial cell proliferation via targeting the vascular endothelial growth factor (Chamorro-Jorganes et al. 2011; Li et al. 2019), miR-342-3p appears to have great potential to repress inflammation in atherosclerosis (Wang et al. 2019) and endothelial NO synthase activity is increased by miR-199a-3p inhibition (Joris et al. 2018). As a result, miRNAs might be, likewise, of relevance as potential biomarkers for various conditions in liquid biopsies as well as therapeutic targets.

Endurance and strength training exercises can change the global transcriptional miRNA patterns (Russell et al. 2013b). Increased amounts of physical activity are associated with altered levels of circulating miRNAs (ci-miRNAs) (Silva et al. 2017). In particular in resistance training, a few miRNAs have been detected that change their expression immediately (miR-143, miR-139, miR-195, miR197, miR-30a, miR-10b, miR-208b, miR-532, miR-133a, miR-133b miR-21, and miR-221), 1 h (miR-206 and miR-181a), 4 h (miR-133a) and 24 h (miR-133b) after a single bout of resistance training (D’Souza et al. 2017; Cui et al. 2017; Vogel et al. 2019). Single bouts of endurance exercises increased miR-1 and miR-133a levels (Nielsen et al. 2010). Three hours after a single bout of endurance training, the expression of miR-1, -133a, -133b and -181a was increased while that of miR-9, -23a, -23b and -31 was decreased (Russell et al. 2013b). Moreover, microRNA-1, -30a and -133a plasma levels were significantly increased in elite and non-elite runners after running a marathon (Clauss et al. 2016). In addition, the ci-miRNAs levels mostly returned to baseline after 24 h, except for slightly increased ci-miR-133a in non-elite runners (Clauss et al. 2016). In the long term, 12 weeks of endurance training lead to a down-regulated levels of several muscle-specific miRNAs (Nielsen et al. 2010).

It is important to note that there are tissue-specific miRNAs (Lagos-Quintana et al. 2002). Not only individual tissues have different miRNA expression, also different training patterns and types potentially lead to different miRNA profiles (Vogel et al. 2019). These miRNA profiles have previously only been considered in relation to strength training; a global screening of miRNAs in response to endurance training lacks in the literature. However, some studies present positive correlations between activity-related parameters and miRNA expression. Furthermore, positive associations between maximal oxygen uptake (VO_2max_) and miR-29a, miR-1 and miR-486 have been reported (Denham and Prestes 2016; Domańska-Senderowska et al. 2017). In addition, there are positive correlations with other exercise-associated parameters such as peak workload, serum creatine kinase (skeletal muscle damage) or changes in levels of high-sensitivity C-reactive protein (an acute-phase inflammatory marker) and miR-221 and miR-146a (Li et al. 2018).

In addition, it appears that many miRNAs can adapt to a specific training type. For example, the specific miRNAs act in the pathways of the appropriate tissues and can influence cardiac/skeletal muscle strength, endothelial function and/or oxygen consumption. One example is the central role of miR-206 in myogenesis. The expression patterns of miR-206 are restricted to skeletal muscle myoblasts and cardiac tissue during embryonic development and muscle cell differentiation (Sweetman et al. 2008). Going further, miR-1 and miR-133a/b, these miRNAs are specifically found in skeletal muscle and can influence several biological processes such as growth, development, atrophy and hypertrophy (Morais et al. 2017). During myogenesis, increased expression patterns of the previously described miRNAs are found and additionally at the same time, skeletal muscle hypertrophy is associated with reduced expression of miR-1 and miR-133a/b (Naguibneva et al. 2006). The association of the reduction of miR-1 and miR-133a and muscle hypertrophy is explained in the literature by the eliminated transcriptional inhibition on growth factors (Huang et al. 2011). They may control myogenesis and regeneration of skeletal muscle mainly through the influence on genes encoding myoblast precursors. Comparably, miR-21 is specific for vascular endothelial cells and appears to be involved in vascular remodeling (Tu et al. 2013; Vogel et al. 2020). However, it is controversial whether the intensity of training or the specific type of training (e.g., endurance or strength training) moderates such specific miRNA effects.

To investigate different training methods and metabolic situations, this study uses blood flow restriction training in addition to low-intensity and high-intensity training endurance training. During training with blood flow restriction, the blood supply to the working muscle is simultaneously throttled and venous return is prevented. Adaptations in the musculature induced by high-intensity training can already be achieved at lower load intensities (Hughes et al. 2017). BFR training can be used in both endurance and strength training (Patterson et al. 2019). Strength training using BFR is shown to improve muscle hypertrophy and strength (Patterson et al. 2019). In addition, positive effects have been found in the endurance domain with gait training in combination with BFR and also with low-intensity cycling training (Abe et al. 2010). However, the metabolic stress and mechanical tension are responsible for a number of mechanisms as primary signaling agents (Patterson et al. 2019). Metabolic stress is thought to be the more dominant stimulus in activating a range of mechanisms (Pearson and Hussain 2015). BFR training is thought to create a hypoxic and acidic environment comparable to exercise with intensities above the anaerobic threshold. Both training stimuli are stressing intramuscular energy metabolism, which is also an important indicator of metabolic stress (Yanagisawa and Sanomura 2017). An increase in lactate and muscle protein synthesis (Fujita et al. 2007) and growth hormone production (Pierce et al. 2006) are mechanisms triggered by BFR training. However, a definitive mechanism for BFR training has yet to be demonstrated.

In addition, no study so far conducted a global screening for endurance related adaptations of miRNA, some studies were already able to report some specific alteration. Russel and colleagues reported that an acute bout of endurance training down-regulates miR-23a expression in skeletal muscle in humans (Russell et al. 2013b). The miR-23a is a direct target for PGC-1α (Russell et al. 2013a), a mitochondrial biogenesis regulator (Olesen et al. 2010). The training-induced change in miRNA expression is a probable muscular adaptation mechanism in response to endurance training. Another example is miRNA-486 which up-regulates insulin-dependent glucose uptake in metabolic tissues, such as skeletal muscles. miRNA-486 is negatively associated with maximum oxygen uptake (VO_2max_) (Small et al. 2010) and thus might be stimulated by endurance exercise. A positive correlation between the levels of ci-miR-29a and VO_2max_ was also noted (Domańska-Senderowska et al. 2017). However, little is known about the interaction of this miRNA within the pathways connected with VO_2_ absorption and consumption. Lastly in mice, miR-696 was postulated as an exercise-dependent regulator of metabolic adaptation (Aoi et al. 2010).

Overall, changes in miRNA expression have great potential for numerous new explanations for the mechanisms of how different exercise types affects human metabolism. The purpose of the study was designed to determine whether the profile of ci-miRNAs is altered after different approaches of single bout of acute endurance exercise (with or without BFR application, as compared with low- and high-volume training protocols with no BFR). Our hypothesis is: low-intensity blood flow single bout of acute endurance exercise and exercise without blood flow restriction lead to different expression patterns of miRNAs.

## Materials and methods

### Ethical standard and study design

The study adopts a randomized-balanced crossover design. Ethical approval was obtained from the local independent institutional review board (protocol number 2018-16, 17.06.2018, Ethics Committee Department 5 Psychology and Sports Sciences Goethe-University Frankfurt). The trial was conducted in accordance with the ethical standards set down by the Declaration of Helsinki (World medical Association Declaration of Helsinki–Ethical Principles for Medical Research Involving Human Subjects) with its recent modification of 2013 (Fortaleza). All participants gave oral and written informed consent prior to study enrollment.

### Sample

Participants were considered eligible if they fulfilled the following criteria: (1) healthy and (2) aged 18–30 years. Exclusion criteria comprised (1) history severe psychiatric, neurological, or cardiovascular diseases; (3) pregnancy; (4) muscle soreness; and (5) intake of analgesics, or muscle relaxants within the previous 48 h.

### Experimental design

The experimental design incorporated three arms. Each participant performed each of the three conditions (on three different days with a washout of at least 7 days in between) in a randomized, balanced sequence. Before the first exercise intervention, blood flow velocity to validate the BFR application and the individual anaerobic threshold on treadmill were determined (please see “Individual anaerobic threshold determination” for details). During each intervention, loading-associated outcomes were monitored. The acute effects were determined using pre- and post-intervention measurements.

### Individual anaerobic threshold determination

All participants carried out a cardiopulmonary exercise testing on a treadmill at a familiarization visit. Based on a modified Balke protocol (start: 5.0 km/h at 7.5% inclination; increase of 1.5% every 3 min) and using the individual lactate values, the individual anaerobic threshold (IANS) according to Dickhuth (Dickhuth et al. 1999) was determined.

### Intervention

The three exercise conditions were (1) single bout of acute endurance exercise without BFR at 100% of the IANS, (2) single bout of acute endurance exercise without BFR at 80% of the IANS, and (3) single bout of acute endurance exercise with BFR at 80% of the IANS. The possible sequences of the conditions to be performed were randomly assigned to the participants in a balanced distribution. Single bout of acute endurance exercise was performed for 20 min on a treadmill (h/*p*/cosmos, Nußdorf, Germany). In the conditions (1) and (2), single bout of acute endurance exercise with 80% of the IANS was adopted. In condition (3), the single bout of acute endurance exercise was performed at 100% of the IANS. During condition (2), blood pressure cuffs (B Strong BFR TRAINING SYSTEM), inflated to 300 mm Hg, were applied to both legs. On each test day, a standardized control condition (“do-nothing” phase) was performed for 20 min before the test condition. The washout phase between the three test days was at least 7 days each time.

### Assessments

#### Laboratory analytic outcomes

##### Blood lactate concentration

Before and directly after each intervention, capillary blood was taken by pricking the earlobe with a safety lancet. The sample was applied directly to a test strip to determine lactate concentration (mmol/L) by means of a portable, hand-held unit (Lactate Scout, SensLab GmbH, Leipzig, Germany).

##### Heart rate

During the intervention, a chest belt (Polar H7) and heart rate receiver (Polar M 430) continuously measured heart rate (beats/min). The maximum heart rate was selected for further analyses.

##### Blood sampling and plasma preparation for miRNA profiling

To minimize pre-analytical variables that might influence the miRNA expression profile, collection of blood and the preparation of plasma were conducted with a minimal risk of blood cell contamination and hemolysis. Before and after each intervention, fingertip capillary blood samples (≥ 200 μL) were collected in microvettes (system for capillary blood collection) containing Ethylenediaminetetraacetic acid (EDTA). Blood samples were centrifuged for 10 min at 3000 rpm and 4 °C. After the first centrifugation step, the upper plasma phase was transferred to a new tube without disturbing the intermediate buffy coat layer. The plasma samples were centrifuged a second time for 10 min at 15,000 rpm and 4 °C. The cleared supernatant was carefully transferred to a new tube and frozen at − 80 °C.

##### Determination of hemolysis

To assess hemolysis, oxyhemoglobin absorbance was measured at 414 nm in plasma samples using NanoDrop (peqlab Biotechnologie GmbH; Erlangen, Germany). Samples with an optical density (OD)_414_ less than 0.3 were selected for the initial screening.

##### miRNA isolation

Sample amounts were standardized by volume: the same volume of plasma was used for each RNA isolation, and the same volume of purified RNA was used for all further analyses. The miRNAs were isolated from 50 µL (qRT-PCR) or 200 µL (PANEL screen) of plasma using a column-based protocol (miRNeasy Serum/Plasma Advanced Kit, (Qiagen, Hilden, Germany) according to the manufacturer’s protocol. cel-miR-39 from *Caenorhabditis elegans* (1 nM) was spiked in. In the final step, total RNA (> 18 nucleotides) was eluted using 20 µL of RNase-free water.

##### Reverse transcription

For reverse transcription, the miRCURY LNA RT Kit (Qiagen, Hilden, Germany) was used. Undiluted complementary DNA (cDNA, 20 µL) was used for miRCURY LNA miRNA Focus Panel Human Serum/Plasma (YAHS-106Y) in the 2 × 96-well plate format. The Human Serum/Plasma Focus Panel includes 179 miRNA assays targeting relevant miRNAs, reference miRNAs, and spike-in controls.

##### Quantitative real-time PCR

Following reverse transcription as described above, quantitative real-time PCR was performed using miRCURY LNA miRNA PCR assays (Appendix Table 1) in a 10-µL reaction containing 3 µL of cDNA (1:30) and a CFX real-time PCR detection system (BioRad, Munich, Germany). Assays were performed in duplicate. The amount of the respective miRNA was normalized to miR-425-3p and cel-miR-39.

For the miRCURY miRNA PCR analysis, v1.0 raw *C*_t_ data from real-time PCR were uploaded at (https://www.qiagen.com/us/shop/genes-and-pathways/data-analysis-center-overview-page). Cel-miR-39-3p was used as an internal spike-in amplification control. A *C*_t_ cutoff of 35 was set as the lower limit of detection. A global *C*_t_ mean of expressed miRNAs was used for normalization, and the fold change was calculated as (2^−ΔΔ*C*t^), which represents the average normalized miRNA expression (2^−ΔΔ*C*t^) of the samples in the test group divided by the average normalized miRNA expression (2^−ΔΔ*C*t^) of the samples in the control group.

#### Self-reported outcomes

Self-reported outcomes consisted of rates of perceived exertion (RPE-Borg; Likert 6 to 20-point scale), current well-being assessments [feeling scale: (+5 to −5, 10 point Likert scale)], and fatigue reporting (numeric rating scale NRS: 0–10 points). All self-reported parameters were assessed once after each intervention. The participants were asked to refer to the highest intensity during (RPE and feeling scale) or at the end (fatigue) of each intervention.

### Data analyses and statistics

For all outcomes assessed before and after each of the exercise bouts, post-exercise values and absolute pre-to-post-differences were used. Variables continuously monitored during the exercise bouts were processed in their real values.

After the plausibility control, all analyses were performed using parametric or non-parametric testing based on the underlying assumptions data structure, distribution of the variances (Kolmogorov–Smirnov-Test with Lilliefors correction) and variance homogeneity (Levene’s Test). Between-group differences and pre-to-post-changes were determined using omnibus and, in case of a significant omnibus main effect, follow-up post hoc testing.

Friedman tests were performed for omnibus between-group comparisons for all exercise bouts values (or the a priori calculated pre-to-post-differences). Significant omnibus testing was followed by post hoc Bonferroni–Holm-alpha-error-adjusted Mann–Whitney *U* tests. For pre-to-post-significance testing, Wilcoxon tests were performed as post hoc analyses.

To identify significant miRNA expression changes between conditions, a fold regulation with a fold-change threshold of 1.4 was calculated. Significant miRNA expression changes were visualized using the volcano plot. For each miRNA showing a significant expression change, a pairwise group comparison (Student’s *t* test) was made based on the 2^−ΔΔ*C*t^ value of the replicate samples. The *p* value calculation was based on a parametric, two-sample, equal variance, unpaired, and two-tailed distribution.

The potential associations between the kinematic (treatment) effects of the miRNA and lactate were analyzed using partial linear regression with a co-variate group allocation.

SPSS 23 (SPSS Inc., Chicago, IL, USA) and GraphPad software PRISM5 for Mac (GraphPad Software, La Jolla, CA, USA) were used to conduct all statistical calculations and create figures. For all statistical analyses, an alpha-error level of 5% was considered a relevant cutoff value for significance testing, *p* values below indicating significant differences/changes/associations.

## Results

### Sample

None of the participants withdrew consent, and no one had to be excluded. Eighteen healthy adults [females = 10; mean age 24, standard deviation (SD) 3 years; body mass index 22.5, SD 2.4 kg/m^2^] were included.

### Basic single bout of acute endurance exercise outcomes

#### Objective outcomes of the interventions

**Fig. 1:**
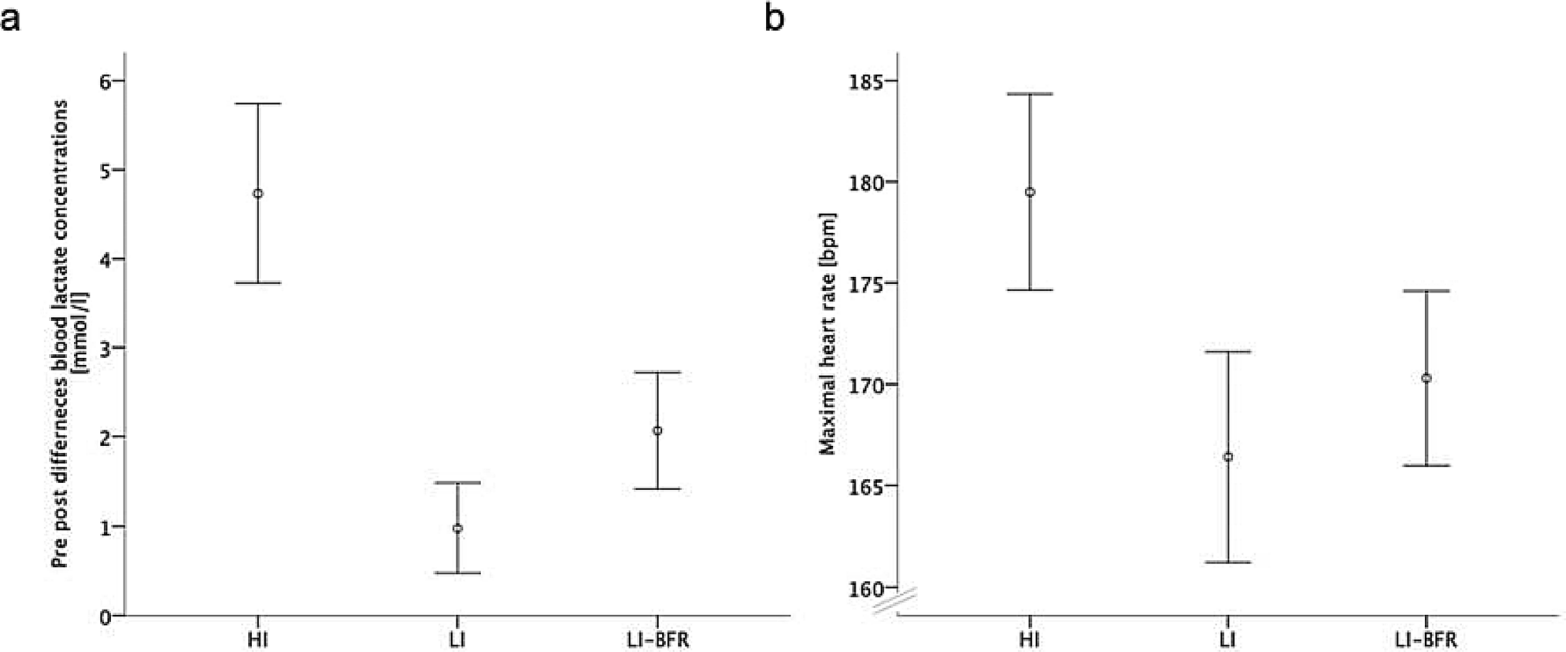
Objective outcomes of training interventions. Data are displayed as mean and 95% confidence intervals. **a** Blood lactate concentration, **b** maximal heart rate. **a** displays the pre-to-post-differences, **b** highlights the real values during the exercises. *Bpm* beats per minute, *LI-BFR* low-intensity exercise with blood flow restriction, *LI* low-intensity exercise, *HI* high-intensity exercise

Two of the objective outcomes increased after all exercise interventions, lactate concentration and heart rate (*p* < 0.001) (Fig. 1). While, in both significant parameters, the high-intensity (HI) intervention resulted in a higher concentration than both lower intensity protocols (*p* < 0.001) and additionally, the low-intensity blood flow restriction (LI-BFR) protocol present a higher heart rate and a higher lactate concentration than the low-intensity (LI) protocol without blood flow restriction (*p* = 0.037; *p* = 0.003).

#### Participant-reported outcomes

**Fig. 2:**
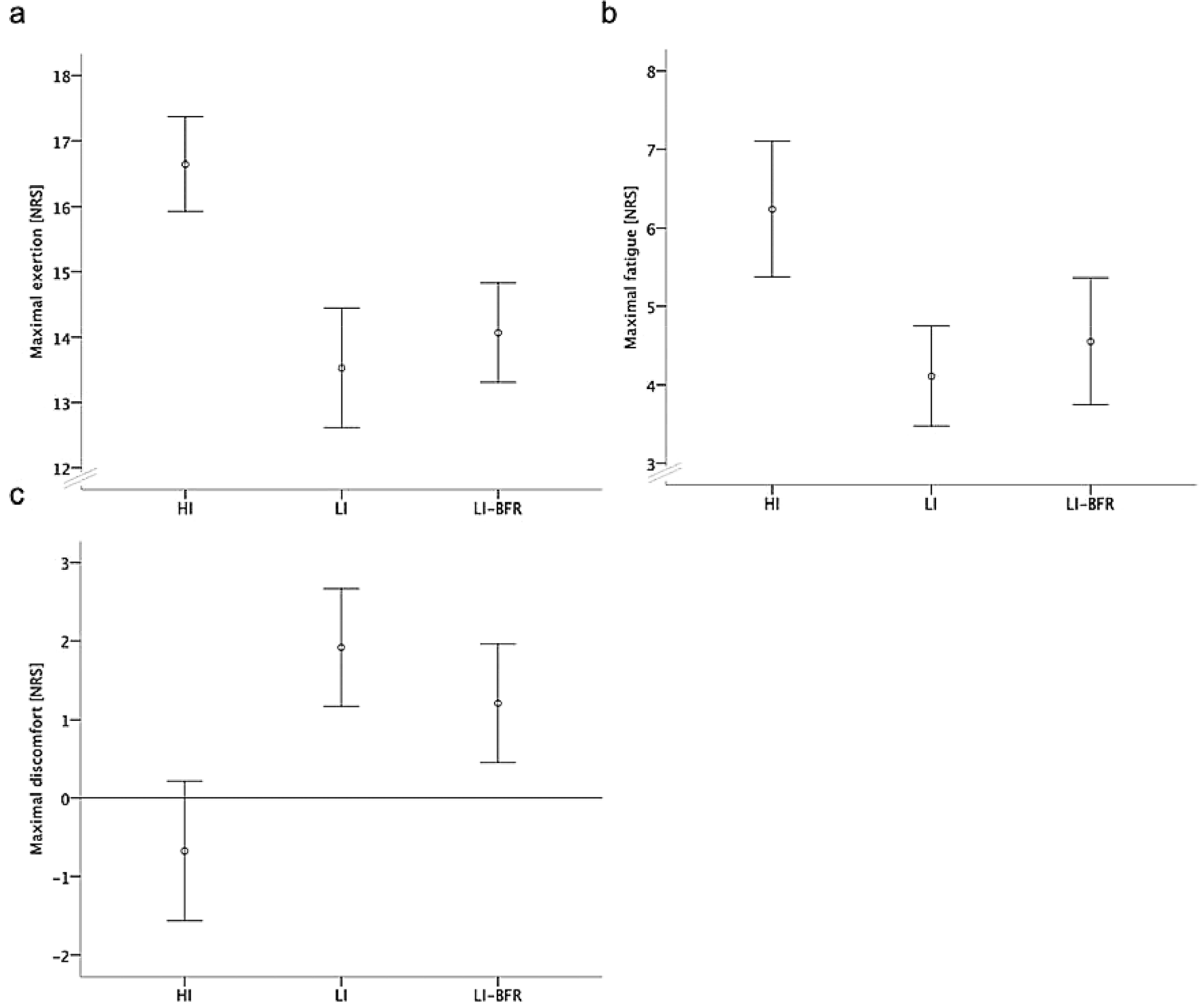
Participant-reported outcomes of training interventions. Data are displayed as mean and 95% confidence intervals. **a** Maximal exertion. **b** Maximal fatigue. **c** Maximal discomfort. **a**, **b** and **c** highlight the real values during the exercises. *NRS* numeric rating scale, *LI-BFR* low-intensity exercise with blood flow restriction, *LI* low-intensity exercise, *HI* high-intensity exercise

The perceived exertion was higher during the HI intervention than during the two LI interventions (*p* < 0.001) (Fig. 2). The HI group reported lower values than both LI groups in the feeling scale (*p* < 0.001). Participants in the HI group reported also higher values in the fatigue scale than the LI-BFR and the LI group (*p* < 0.001). The LI-BFR group showed higher values in the fatigue than the LI intervention without BFR (*p* = 0.054).

### Profiling of circulating miRNAs

#### Screening of expression changes in circulating miRNAs before and after high intervention exercise

**Fig. 3:**
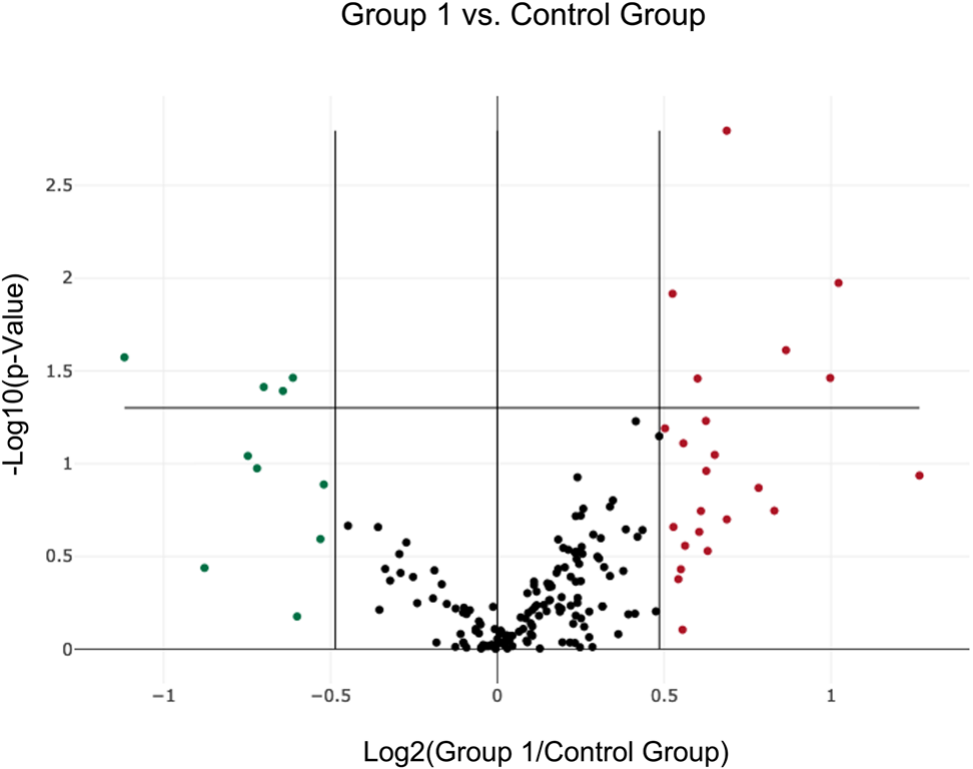
Volcano plot of differentially expressed miRNAs pre- and post-HI training. Data points outside the vertical lines are up-regulated (red) or down-regulated (green) more than 1.4-fold. Data points above the solid horizontal line have *p* values less than 0.05

Eight plasma samples from four participants were selected for miRNA screening based on a sample OD_414_ < 0.3 and a lactate difference > 4 before (control) and after training (HI). These were subjected to the human serum/plasma focus panel consisting of 179 miRNA assays. The panel targets human plasma-relevant miRNAs, reference miRNAs and spike-in controls. The use of a global *C*t mean of expressed miRNAs was used for normalization and in addition, cel-miR-39-3p was included as an internal amplification control. In each sample, more than 80% of miRNAs exceeded the lower detection limit of a *C*t < 35 (data not shown). Significant miRNA expression changes were visualized in the volcano plot (Fig. 3). A total of 10 miRNAs were selected for further validation based on their significantly changed expression (fold difference > 1.4; *p* value < 0.05) and analyzed in all intervention groups.

#### Analysis of miRNAs in different training intervention groups

**Fig. 4:**
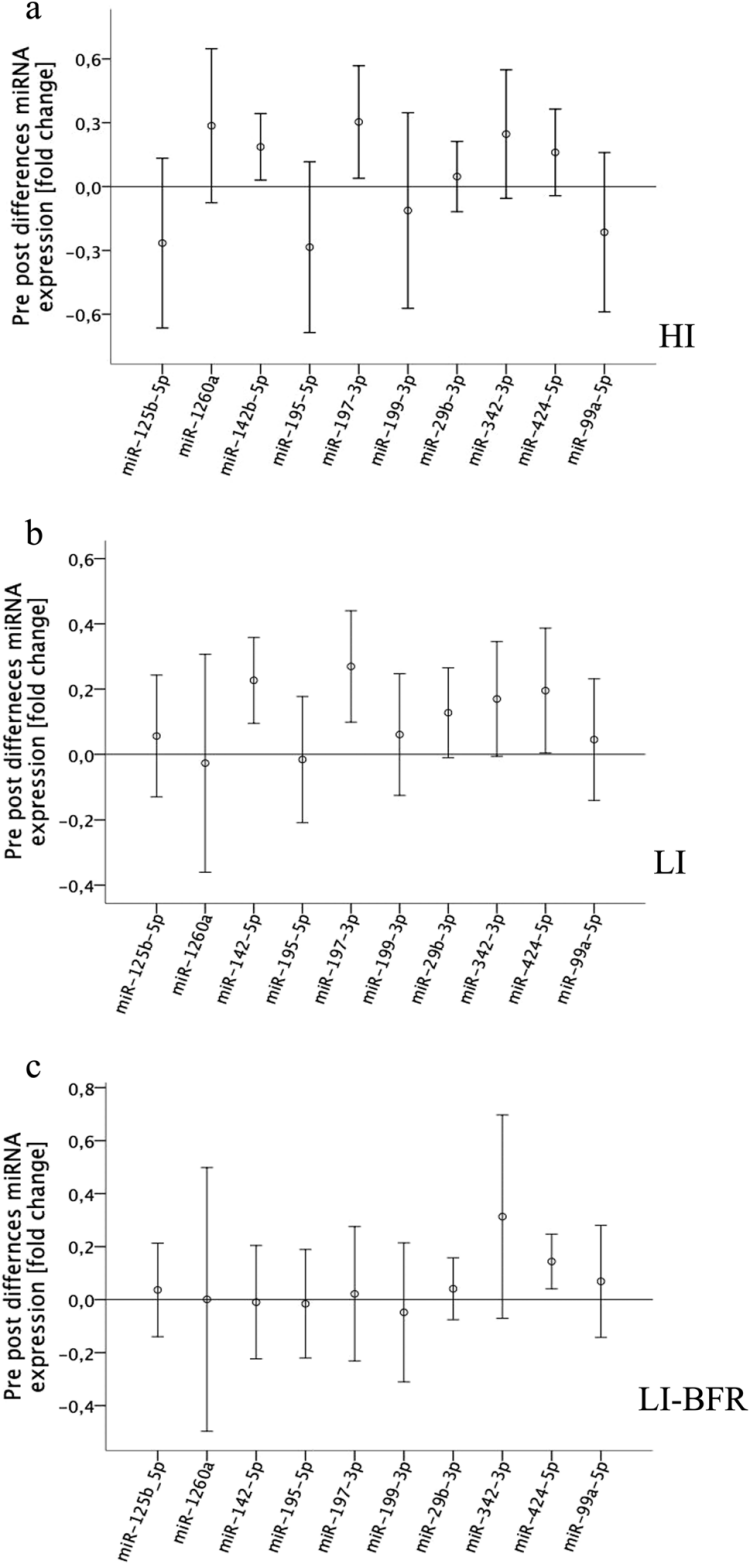
Differences in miRNA expression pre-and post-training. Data are displayed as mean and 95% confidence intervals. **a** *HI* high-intensity exercise. **b** *LI* low-intensity exercise. **c** *LI-BFR* low-intensity exercise with blood flow restriction

The abundance of these 10 differentially expressed miRNAs was analyzed in each exercise group in individual assays in a larger cohort of 18 participants. In the HI and the LI exercise, miR-142-5p and 197-3p were up-regulated (*p* < 0.05). In addition, after the LI exercise, the expression of miR-342-3p was up-regulated (*p* = 0.039). MiR-342-3p and miR-424-5p was confirmed to be up-regulated after LI-BFR exercise (*p* < 0.05) (Fig. 4).

### Associations between exercise outcomes and individual circulating miRNAs

**Fig. 5:**
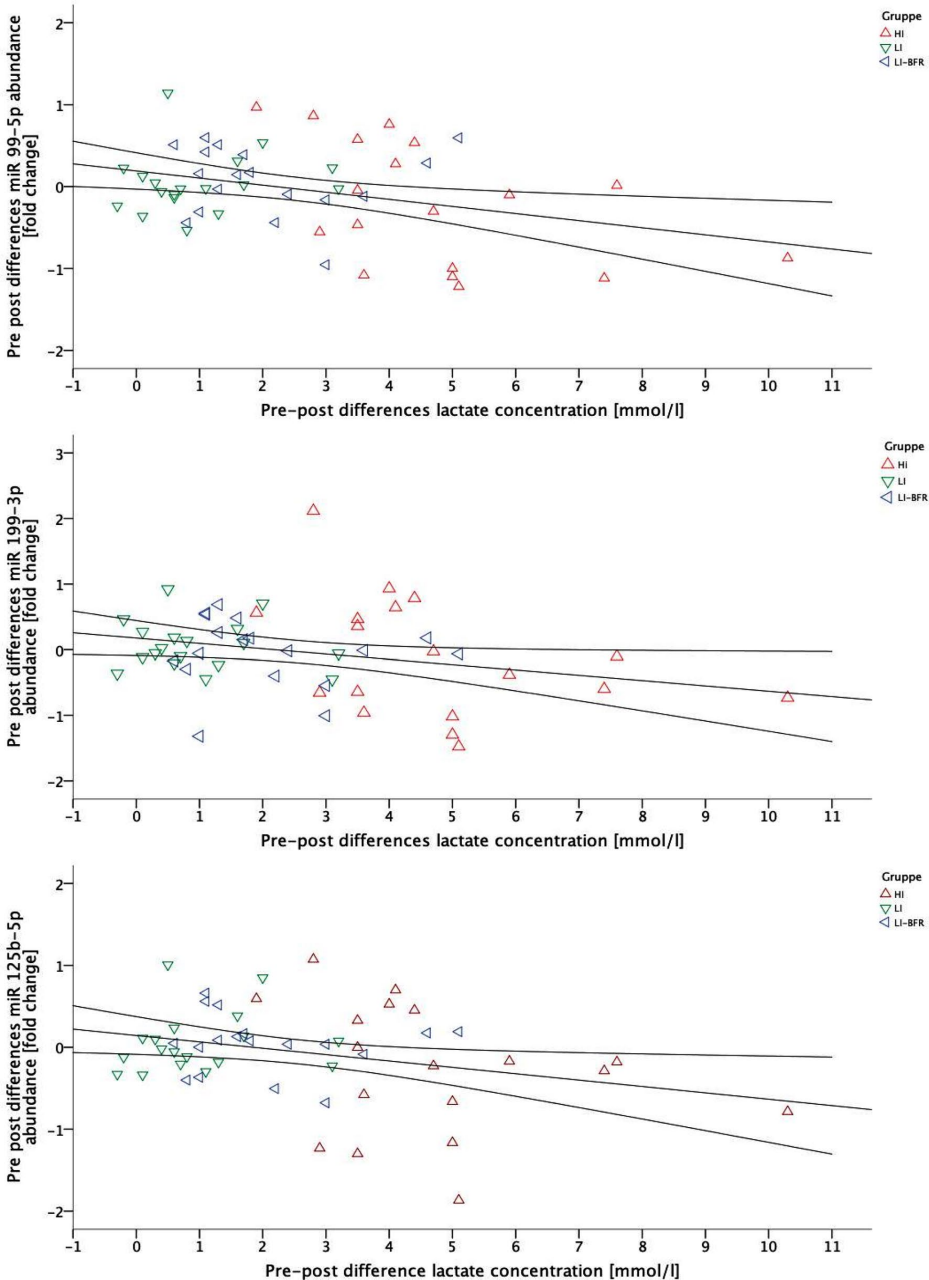
Scatterplot diagrams of **a** miR-99-5p, **b** 199-3p, **c** miR-125b-5p and lactate concentration with a correlation line (including confidence intervals); *HI* high-intensity exercise, *LI* low-intensity exercise, *LI-BFR* low-intensity exercise with blood flow restriction

The pre-to-post-changes of the lactate concentration and the three miRNA levels were significantly linear and negative correlated (miR-99-5p, *p* = 0.011, *r = − *0.343; miR-199-3p, *p* = 0.045, *r = − *0.274 and miR-125-5p, *p* = 0.026, *r = − *0.302). Considering the type of exercise as partializing co-variate, lactate was significant linear negative correlated to miRNA-99-5p (*p* = 0.042 and *r = − *0.280) and miR-199-3p (*p* = 0.033 and *r = − *0.293). The correlations are visualized in Fig. 5. No other systematic correlation between lactate concentration and miRNA expressions occurred (each *p* > 0.05).

## Discussion

In this three-armed crossover study, we investigated the miRNA profiles elicited by three different acute single bout of acute endurance exercise interventions without or with peripheral blood flow application (HI, LI, LI-BFR) in young, healthy athletes. We were able to confirm different miRNA profile adaptations of exercise with characteristic BFR compared to exercise without BFR.

All interventions induced increases in lactate and heart rate; the largest effects on lactate and heart rate occurred after the HI intervention, the LI-BFR group showed lager effects in lactate and heart rate than the LI intervention group. The LI intervention resulted in the smallest pre-to-post-intervention differences in both the objective and participant-reported outcomes. The metabolic response thus increases in relation to exercise intensity.

Current recommendations for resistance exercise constitute that training effects of 70% 1RM intensity without BFR are similar to 30% 1RM with BFR (Loenneke and Pujol 2009). In the present study, we transferred this assumption to single bout of acute endurance exercise.

Based on our findings, we cannot support the assumption that BFR was feasible to induce metabolic stress comparable to endurance exercise at the anaerobic threshold. Since the applied intensity during HI exercise was intended to reach high lactate production levels and exceed energy expenditure which can be obtained on aerobic pathways, it is likely that this training induced the largest stimulus based on hypoxic and acidic environment. The finding of a BFR-induced increase in lactate concentration in two training sessions of comparable intensity is consistent with previous reports (Vogel et al. 2019), although the effects of BFR training were lower than the high-intensity training session. This is in contrast to other studies using BFR, however, the majority of current studies investigated BFR training using an older or untrained study population (Formiga et al. 2020; Minniti et al. 2020). In the present study, a very active and already endurance-trained population was investigated. Current literature suggests that for this study population, higher intensity BFR training should be used to provide an adequate training stimulus and achieve the metabolic response (Minniti et al. 2020). One other reason may be the transfer from resistance training to endurance training. Only a small number of studies used blood flow restriction in endurance training (Minniti et al. 2020). In addition, a study limitation is the use of capillary blood. In the most studies with ci-miRNA, venous blood is used to avoid low blood volume and hemolytic blood. Further on, we only investigated a small but homogenous sample size.

For miRNA profiling, we included plasma samples of four participants with an OD_414_ < 0.3 and an increased lactate concentration (difference > 4) after intervention, a maximal heart rate during exercise of at least 60% of maximal calculated heart rate, and a participant-reported “very hard” intensity on the Borg scale of at least 16 points (data not shown) and screened 179 ci-miRNAs before and after high-intensity acute single bout of acute endurance exercise. Our results of the quantification in each intervention group of the 10 differentially expressed miRNAs before and after exercise suggest that the profile of ci-miRNAs is altered as an acute effect of acute single bout of acute endurance exercise. In particular, we identified two miRNAs (miR-142b-5p and miR-197-3p) that are up-regulated after a single bout of acute endurance exercise without blood flow restriction. In addition, in both LI exercise sessions, miR-342-3p is up-regulated. Only miR-424-5p was found to be up-regulated after blood flow restriction (LI-BFR) exercise. Other studies also demonstrated both acute effects of intensive stimuli (miR-10b, miR-21, miR-30a, miR-139, miR-143, miR-146a, miR-195, miRN-197, miR-221, and miR-222) and exercise effects (miRNA-146a, miRNA-222, and miRNA-20a) (Baggish et al. 2011; Vogel et al. 2019) on the expression of ci-miRNAs. Consequently, we conclude that miRNA-197 is up-regulated through physical activity in general (different sessions of resistance and single bout of acute endurance exercise). In contrast, miR-142-5p could be only triggered in acute single bout of acute endurance exercise and miRNA-342 only in low-intensity single bout of acute endurance exercise. In addition, miRNA-424-5p could represent a unique feature of the single bout of acute endurance exercise with blood flow restriction.

The correlation of the lactate difference (BFR-LI, LI, and HI) and three detected miRNAs (miR-99-5p, miR-199-3p, and miR-125b-5p) abundance further indicates an association with exercise intensity. Consequently, not only the lactate concentration (or the intensity of the exercise), is decisive for miRNA expression—as is the type of exercise. More concretely, BFR not only leads to lower lactate increases but also tendentiously leads to a different expression of miRNAs. Whether the differences between the conditions are due to the lower intensity or the type of exercise may be finally delineated using an increased intensity during BFR in trained populations, as described above.

It is still unclear to what extent ci-miRNA expression is altered by physical activity and which mechanisms are behind these changes. There are some regulatory mechanisms that are suspected as explanatory approaches, but these mechanisms appear to be very complex and are not yet fully understood (Gulyaeva and Kushlinskiy 2016; Vogel et al. 2020). Two possible mechanisms have been linked to skeletal muscle, one being selective uptake by skeletal muscle during exercise and the other being the release of certain miRNAs from skeletal muscle. With regard to the selective uptake of ci-miRNAs, several studies show that this ability is exercised by target cells, such as skeletal muscle (Vickers et al. 2011; Mittelbrunn et al. 2011). Similarly, there are already some studies showing that physical activity can cause the release of certain miRNAs from skeletal muscle, e.g., due to muscle damage or as a general response to acute or regular exercise (Aoi et al. 2013; Denham and Prestes 2016).

The detected miRNAs have already been linked to different cardiovascular processes before. For example, miRNA-142-5p is associated with the VEGF and mTOR signaling pathways. This miRNA could be a factor in hypertrophy and improvement of the cardiovascular system, endurance performance and thus possibly objective factors such as VO_2max_. So far, most studies have associated this miRNA with cardiac muscle. In addition, up-regulation of miR-142-5p expression is associated with apoptosis in human macrophages through targeting TGF-β2, this effect may play an important role in the progression of atherosclerosis (Xu et al. 2015).

MiR-197-3p seems to be regulated independent of the type of exercise (Vogel et al. 2019). A predictive value of miR-197-3p is discussed as association with the incidence of myocardial infarction (Zampetaki et al. 2012).

miRNA-342-3p is linked to the immune system after a single exposure of physical activity and is often found in neutrophil cells and circulating blood (Rutledge et al. 2015). A change in this miRNA was only found in low-intensity exercise interventions in the present study. Some studies present that acute bouts of exercise are able to modulate metabolic-endocrine parameters (Antunes et al. 2020). In general, aerobic exercise appears to be more effective in altering the immune system and inflammatory markers (Abd El-Kader and Al-Shreef 2018) than anaerobic exercise.

MiR-424-5p has been found to be associated in post-ischemic vascular remodeling and angiogenesis. It has also been reported that miR-424-5p regulates hypoxia-inducible factor (HIF)-α isoforms and promotes angiogenesis (Ghosh et al. 2010). This is in line with our observation that only blood flow restriction exercise stimulated the expression of this particular miRNA. This finding is supported by studies reporting that BFR resistance training improves vascular endothelial function and peripheral blood circulation with increased expression of lactate, noradrenaline, vascular endothelial growth factor and growth hormones (Shimizu et al. 2016).

Our results are consistent with the proposed epigenetic potential of lifestyle interventions that may alter gene expression (Domańska-Senderowska et al. 2017; Barber et al. 2019). Therefore, we postulate that further studies are necessary to adjust the intensity and extent of training to gain more information about the relationship between individual miRNAs and human metabolism. This could be an added value (1) in training control and (2) in preventive intervention screening of miRNAs to stratify responders and non-responders for individualization of intervention/training goals and (3) the use of suitable training stimuli in rehabilitation (strength training vs. endurance training; HI training vs. BFR training) based on metabolic changes in the miRNA expression.

## Conclusion

In conclusion, it appears that single bout of acute endurance exercise as well as exercise induces changes in the ci-miRNA expression. These changes are during short periods of single bout of acute endurance exercise without blood flow restriction as well as over short periods of single bout of acute endurance exercise with blood flow restriction. Future studies are necessary to identify whether these changes in miRNAs play a physiological role by which endurance exercise affects whole-body metabolism.

## Appendix

See Table 1

**Table 1:** miRCURY LNA miRNA PCR assays for screening hits

hsa-miR-197-3p	YP00204380	5′UUCACCACCUUCUCCACCCAGC
hsa miR-342-3p	YP00205625	5′UCUCACACAGAAAUCGCACCCGU
hsa-miR-424-5p	YP00204736	5′CAGCAGCAAUUCAUGUUUUGAA
hsa-miR-29b-3p	YP00204679	5′UAGCACCAUUUGAAAUCAGUGUU
hsa-miR-1260a	YP00205892	5′AUCCCACCUCUGCCACCA
hsa-miR-142-5p	YP00204722	5′CAUAAAGUAGAAAGCACUACU
hsa-miR-99a-5p	YP00204521	5′AACCCGUAGAUCCGAUCUUGUG
hsa-miR-199a-5p	YP00204494	5′CCCAGUGUUCAGACUACCUGUUC
hsa-miR-125b-5p	YP00205713	5′UCCCUGAGACCCUAACUUGUGA
hsa-miR-195-5p	YP00205869	5′UAGCAGCACAGAAAUAUUGGC

## Abbreviations

bFGF: Basic fibroblast growth factor
BFR: Blood flow restriction
BPM: Beats per minute
ci-miRNA: Circulating microRNA
EDTA: Ethylenediaminetetraacetic acid
FSS: Fluid shear stress
HI: High intensity
HR: Heart rate
IANS: Individual anaerobic threshold
LC: Lactate concentration
LI: Low intensity
LI-BFR: Low intensity with blood flow restriction
miRNA: MicroRNA
mTOR: Mechanistic target of rapamycin
NRS: Numeric rating scale
OD: Optical density
PAD: Peripheral artery disease
PCR: Polymerase chain reaction
RNA: Ribonucleic acid
RPE: Rates of perceived exertion
SD: Standard deviation
UTR: Untranslated region
VEGF: Vascular endothelial growth factor
VO2: Oxygen uptake
VO_2max_: Maximal oxygen uptake
